# Effect of chitooligosaccharides with a specific degree of polymerization on multiple targets in T2DM mice

**DOI:** 10.1186/s40643-022-00579-3

**Published:** 2022-09-05

**Authors:** Jiangshan You, Mengyao Zhao, Shumin Chen, Lihua Jiang, Shuhong Gao, Hao Yin, Liming Zhao

**Affiliations:** 1 State Key Laboratory of Bioreactor Engineering, School of Biotechnology, East China University of Science and Technology, Shanghai, 200237 China; 2 Organ Transplant Center, Shanghai Changzheng Hospital, Shanghai, 200003 China; 3 Shanghai Collaborative Innovation Center for Biomanufacturing Technology (SCICBT), Shanghai, 200237 China

**Keywords:** Chitooligosaccharides, Diabetes mellitus, Insulin resistance, Hypoglycemic effect

## Abstract

Chitooligosaccharides (COS) are found naturally in the ocean and present a variety of physiological activities, of which hypoglycemic action has attracted considerable research attention. This study aimed to assess the therapeutic effect of COS on mice suffering from type 2 diabetes mellitus (T2DM). COS effectively reduced blood glucose and blood lipid levels and improved glucose tolerance. Furthermore, COS revealed strong inhibitory activity against α-glucosidase, reducing postprandial blood glucose levels. Molecular docking data showed that COS might interact with surrounding amino acids to form a complex and decrease α-glucosidase activity. Additionally, COS enhanced insulin signal transduction and glycogen synthesis while restricting gluconeogenesis in the liver and muscles, reducing insulin resistance (IR) as a result. Moreover, COS effectively protected and restored islet cell function to increase insulin secretion. These results indicated that COS exhibited a significant hypoglycemic effect with multi-target participation. Therefore, COS may serve as a new preventive or therapeutic drug for diabetes to alleviate metabolic syndrome.

**Graphical Abstract:**

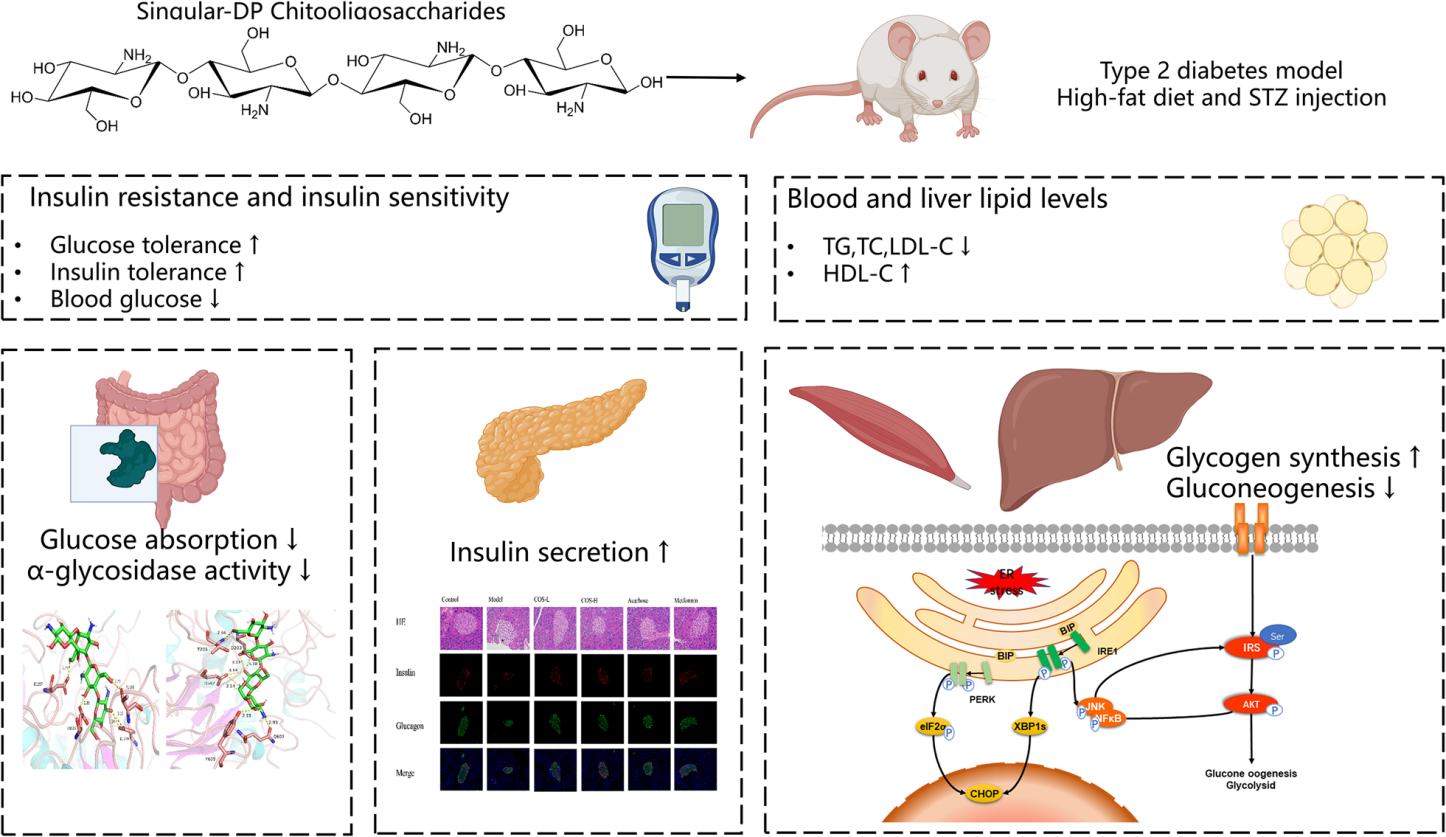

**Supplementary Information:**

The online version contains supplementary material available at 10.1186/s40643-022-00579-3.

## Introduction

Type 2 diabetes mellitus (T2DM) represents a chronic metabolic disease, widely recognized as a serious global health concern (Federation 2019). An estimated 415 million adults suffered from this disease in 2019, which is anticipated to increase to 642 million by 2040 (Federation 2019). T2DM is characterized by beta-cell inefficiency and insulin resistance (IR) accompanied by high blood glucose levels (Zheng et al. 2018). Therefore, controlling the blood glucose levels of diabetic patients, improving IR, and restoring pancreatic beta-cell functionality are effective approaches to treating diabetes (Samuel and Shulman 2012; Meo et al. 2017; Yaribeygi and Butler 2019). Several classes of anti-diabetic medications are currently available. Acarbose inhibits alpha-glucosidase activity and controls postprandial glucose levels in the blood, and biguanides reduce glucose absorption and utilization by the liver and skeletal muscles. Thiazolidinediones increase insulin sensitivity, while sulfonylureas and glinides promote insulin secretion by pancreatic islet cells (Vieira et al. 2019). However, these drugs present limitations, such as a lack of a multi-organ synergistic effect, as well as undesirable side effects and toxicity. Therefore, natural products have gained considerable popularity due to their efficacy and chronic disease conditioning while displaying fewer side effects and a higher level of safety. Consequently, it is necessary to derive safer, more effective, and more economical therapeutic agents for diabetes from food.

Blood glucose balance is typically affected by various factors, such as controlling intestinal carbohydrate absorption after a meal to reduce postprandial hyperglycemia and the beta-cell regulation of metabolic homeostasis via insulin secretion into islet capillaries after increased blood glucose sensing. Additional influencing factors include the absorption and utilization of glucose from the target liver tissue and muscles (Zhang et al. 2013; Salvadó et al. 2015), while the IRS/Akt pathway plays a vital role in glucose utilization. A damaged IRS/Akt pathway causes or aggravates IR, leading to serious issues, such as glucose transport dysregulation, gluconeogenesis, and glycogen synthesis (Bathina and Das 2018; Reda et al. 2018). Additionally, endoplasmic reticulum (ER) stress is considered a leading factor in metabolic insulin signaling (Mathijs et al. 2014; Duan et al. 2017; Garner et al. 2018; Lee et al. 2018; Coker‐Gurkan et al. 2019; Liu et al. 2019). Insulin signaling in the peripheral tissues can be restricted by ER stress via the activation of signaling cascades, such as eukaryotic translation initiation factor 2 (elF2α) and c-Jun N-terminal kinase (JNK) (Zhang et al. 2013; Salvadó et al. 2015). Therefore, reducing glucose uptake and ER stress, increasing glucose utilization, and facilitating insulin signal transduction are essential for improving and treating diabetes.

Chitooligosaccharides (COS) are naturally found in the ocean and are typically acquired via the complex hydrolysis of shrimp and crab shells (Naveed et al. 2019). They present advantages, such as low cost, exceptional water solubility, and high bioavailability. Furthermore, COS displays various bioactivities (Aam et al. 2010; Katiyar et al. 2011; Oligosaccharides 2015; Muanprasat and Chatsudthipong 2017), of which its hypoglycemic properties are widely recognized (Yu et al. 2017; Bai et al. 2018; Li et al. 2018; Zhao et al. 2019, 2020; Deng et al. 2020). In vitro evaluations and clinical experiments have demonstrated that COS reduces postprandial blood glucose levels and improves IR (Ju et al. 2010; Jo et al. 2013, 2014; Kim et al. 2014). However, previous studies have primarily used COS mixtures, while the structural characteristics remain unclear. Therefore, the specific COS structure requires clarification to investigate the intervention mechanism.

This study aims to elucidate the hypoglycemic activity of COS with a well-defined degree of polymerization (DP). Furthermore, the potential therapeutic impact of COS on T2DM is evaluated using multiple in vivo targets (small intestine, pancreas, liver, and muscles) to further investigate the role of the hypoglycemic COS mechanism with a single DP while considering multi-organ coordination for controlling blood glucose. Furthermore, a combination of computational molecular simulations based on structural model calculations and docking was used to clarify the molecular mechanism underlying the hypoglycemic effect of specific singular-DP COS. These results promote the examination of the relationship between the chemical structure and activity of functional oligosaccharides for developing new bioactive anti-diabetic substances.

## Materials and methods

### Materials

The COS was obtained via chitosan degradation using an enzyme–membrane coupling reactor system reported in previous studies with some modifications (Qin et al. 2018). Affinity (Cincinnati, OH, USA) provided the mouse monoclonal anti-p-eIF2α (Ser52), p-IRS1 (Ser307), and p-JNK (Thrl83/Tyrl85) antibodies. Cell Signaling Technology (Beverly, MA, USA) provided the anti-Bax, Bcl-2, BIP, CHOP, NF-κB (p-p65), p-AKT (Ser473), cleaved-PARP, cleaved-caspase-9, cleaved-caspase-3, and GAPDH mouse monoclonal antibodies. The qPCR reagents were purchased from Vazyme (Nanjing, China), while the Shanghai Jining Co., Ltd. (Shanghai, China) and Nanjing Jiancheng Bioengineering Institute (Nanjing, China) provided the mouse enzyme-linked immunosorbent assay (ELISA) kits. The commercial kits with standard protocols were acquired from the Nanjing Jiancheng Bioengineering Institute (Nanjing, China).

### COS characterization

The final COS product quantitation and characterization were performed using MALDI-TOF-MS (a 4700 Proteomics Analyzer; Applied Biosystems, Foster City, CA) and an HPLC system equipped with an ELSD detector (Shimadzu 20A; Shimadzu, Kyoto, Japan).

### Animals and treatment

The Animal Care and Use Committee of Laboratory Animals provided research ethics approval. The specific-pathogen-free (SPF) grade C57BL/6 mice were provided by the Shanghai Jihui Experimental Animal Feeding Co., Ltd (Shanghai, China, Animal Certificate Number: 20170012004449). The mice were raised at the Chengqin Biotechnology (Shanghai) Co., Ltd (License number: SYXK (HU) 2019-0013).

The mice had free access to water and food and were divided into six groups (*n* = 8 per group). The standard diet (Research Diet D12450J) group (Control) was given a daily saline dose as solvent control. The remaining groups received a high-fat diet (HFD) (Research Diet D12492) containing 20% protein, 20% carbohydrate, and 60% fat. After an 8-week breeding period, the mice were inspected for glucose tolerance. Streptozotocin (STZ) dissolved in 0.1 mol/L sodium citrate–hydrochloric acid buffer (at pH = 4.5) was injected daily at a low 35 mg/kg dose over the next 5 days. The glucose levels in the blood of the mice were measured randomly after STZ injection at 3 days, 7 days, and 10 days using a glucometer. The model was established successfully when the blood sugar level exceeded 16.7 mmol/L.

**Fig. 1:**
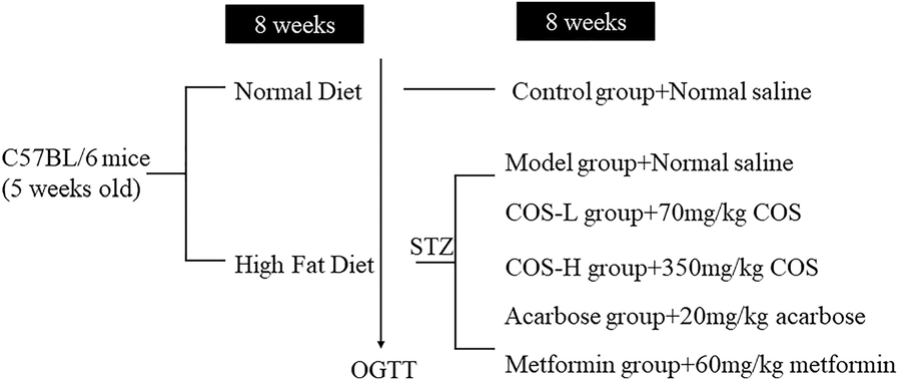
Grouping of the animal experiments

Acarbose, metformin, and COS were dissolved in 0.9% NaCl, different doses of which were intragastrically administered to the intervention groups. The groups and dosages are shown in Fig. 1. All the mice were killed after 8 weeks and a fasting period of 18 h.

### The insulin tolerance test (ITT) and oral glucose tolerance test (OGTT)

The ITT and OGTT were conducted at the end of the administration cycle. The mice were subjected to an overnight fasting period of 12 h, after which they were given a 2 g/kg glucose dose. The glucose levels in the blood samples obtained from the tail veins were evaluated at 0 min, 15 min, 30 min, 60 min, 90 min, and 120 min using a Roche glucometer (Germany). The ITT was performed after a one-week recovery period. The mice were fasted for 6 h, after which they received an injection of 1 unit of insulin per 1 kg into the peritoneum. The subsequently collected blood samples were used for blood glucose level assessment. The area under the curve (AUC) was measured to quantify the OGTT and ITT.

### Serum and tissue collection

Clean 1.5 mL Eppendorf tubes were used to collect blood from the intra-orbital venous plexus. The blood samples were stored overnight at 4 °C and centrifuged at 106*g* for 15 min, after which the supernatant was collected using clean EP tubes and stored at − 80 °C. The livers, pancreas, epididymal fat, femoral skeletal muscles, hearts, spleens, and kidneys were dissected and weighed. The livers, fat, muscles, pancreas, small intestines, and spleens of each group were cryopreserved and fixed with paraformaldehyde. Additional samples were collected and stored at − 80 °C for subsequent detection.

### Biochemical analysis of the serum and liver

Enzyme-linked immunosorbent assay (ELISA) kits were used to determine the insulin and glycosylated hemoglobin levels in the serum of the mice. The homeostasis model assessment for IR (HOMA-IR) index was calculated using the following formula: fasting insulin value (μU/mL)/22.5 × fasting blood glucose value (mmol/L).

The total triglyceride (TG), total cholesterol (TC), low-density lipoprotein (LDL-C), and high-density lipoprotein (HDL-C) levels in the serum and livers of the mice were determined using commercial kits obtained from the Nanjing Institute of Bioengineering (Nanjing, China). The total protein was normalized according to the results and determined using a Bradford protein assay kit (Bio-Rad, Hercules, CA).

### Glycogen quantification

The glycogen content in the muscle and liver tissues was measured using glycogen determination kits acquired from the Nanjing Jiancheng Bioengineering Institute (Nanjing, China) as per the instructions of the manufacturer.

### Intestinal glycosidase activity

Fresh duodenal content was collected immediately after dissection. This material was diluted at different proportions, homogenized mechanically using an ice-water bath, and centrifuged at 1300*g* for 10 min using a refrigerated centrifuge. The supernatant was subsequently collected, and the digestive enzyme activity was tested according to the instructions of the manufacturer.

### Histopathological examination

The liver, muscle, small intestine, and pancreatic tissues obtained from each group of mice were fixed in a 10% paraformaldehyde solution, dehydrated with graded ethanol, and placed in paraffin. Then, 5-μm sections of the samples were stained with hematoxylin and eosin (HE), after which the histopathological changes were monitored using a vertical optical microscope (Nikon Eclipse CI, NIKON, Japan).

### Immunohistochemical assessment of the mouse pancreas

The pancreatic paraffin sections were dewaxed and dehydrated. The tissue slices were incubated overnight with the primary IR (Servicebio, GB11334, 1:300) and glucagon resistance (Servicebio, GB13097, 1:100) antibodies at 4 °C. The tissue slices were then washed and incubated at room temperature for 1 h with Alexa Fluor 488-conjugated goat anti-rabbit IgG (Servicebio, GB25303, 1:400) and Alexa Fluor Cy3-conjugated goat anti-mouse IgG (Servicebio, GB21303, 1:300). The slices were rewashed and stained with DAPI for 10 min at room temperature. A confocal fluorescence microscope (Ti-U, NIKON, Japan) was used to obtain images of the slices.

### Real-time fluorescence quantitative PCR

The total RNA in the mouse tissues was acquired using an extraction RNA kit (Vazyme Biotech Co. Ltd., Nanjing, China) according to the instructions of the manufacturer, after which the absorbance was measured at 260/280 nm to determine the quantity and purity of the RNA. Then, cDNA was synthesized from the total RNA using a reverse transcription kit obtained from the Vazyme Biotech Co. Ltd. (Nanjing, China). Next, qPCR was performed using SYBR Premix Ex Taq (Vazyme Biotech Co. Ltd., Nanjing, China) according to the instructions of the manufacturer. The detected mRNA-to-GAPDH ratio was determined with GAPDH considered an internal control. Additional file 1: Table S1 provides the target gene primers.

### Western blot

The liver, muscle, and pancreatic samples were homogenized in a lysis buffer. A mixture of phosphate and protease inhibitors was then dissolved in the lysis buffer, after which the lysate protein concentrations were identified using a BCA protein detection kit. Then, 10% sodium dodecyl sulfate (SDS)-polyacrylamide gel electrophoresis was used to separate an equal amount of protein, which was transferred to a polyvinylidene fluoride (PVDF) membrane obtained from GE Healthcare (Buckinghamshire, UK). This membrane was subjected to incubation for 1 h at room temperature in a blocking agent containing 5% skim milk powder or bovine albumin (BSA), after which it was further incubated at 4 °C with specific primary antibodies, such as GAPDH (1:3000 dilution), p-eIF2α (1:1000 dilution), p-JNK (1:1000 dilution), BIP (1:1000 dilution), p-Akt (1:1000 dilution), and p-IRS1 (1:1000 dilution). The membrane was then incubated at room temperature for 1 h with the secondary antibody, after which the blot was developed using a chemiluminescence (ECL) detection kit. Finally, the intensity of the bands was quantified via grayscale analysis.

### Molecular docking

Chem 3D software was used to construct the molecules, while the compound configuration was optimized via MM2 molecular mechanics. The three-dimensional protein structure is available in the RCSB Protein Data Bank (www.rcsb.org) for the proteins used in this study (PDB Codes: 3TOP, 2QMJ, 1GZO). Autodock Vina 1.1.2 was employed for semi-flexible docking, while Pymol and LigPlot were used for plotting.

### Statistical analysis

GraphPad Prism 6 mapping software was used for plotting. The experimental data were expressed as mean ± standard deviation (SD). The differences between the groups were determined using one-way analysis of variance (ANOVA), Duncan’s test, and SPSS software, while *p* < 0.05 was deemed statistically significant.

## Results

### COS characterization

**Fig. 2:**
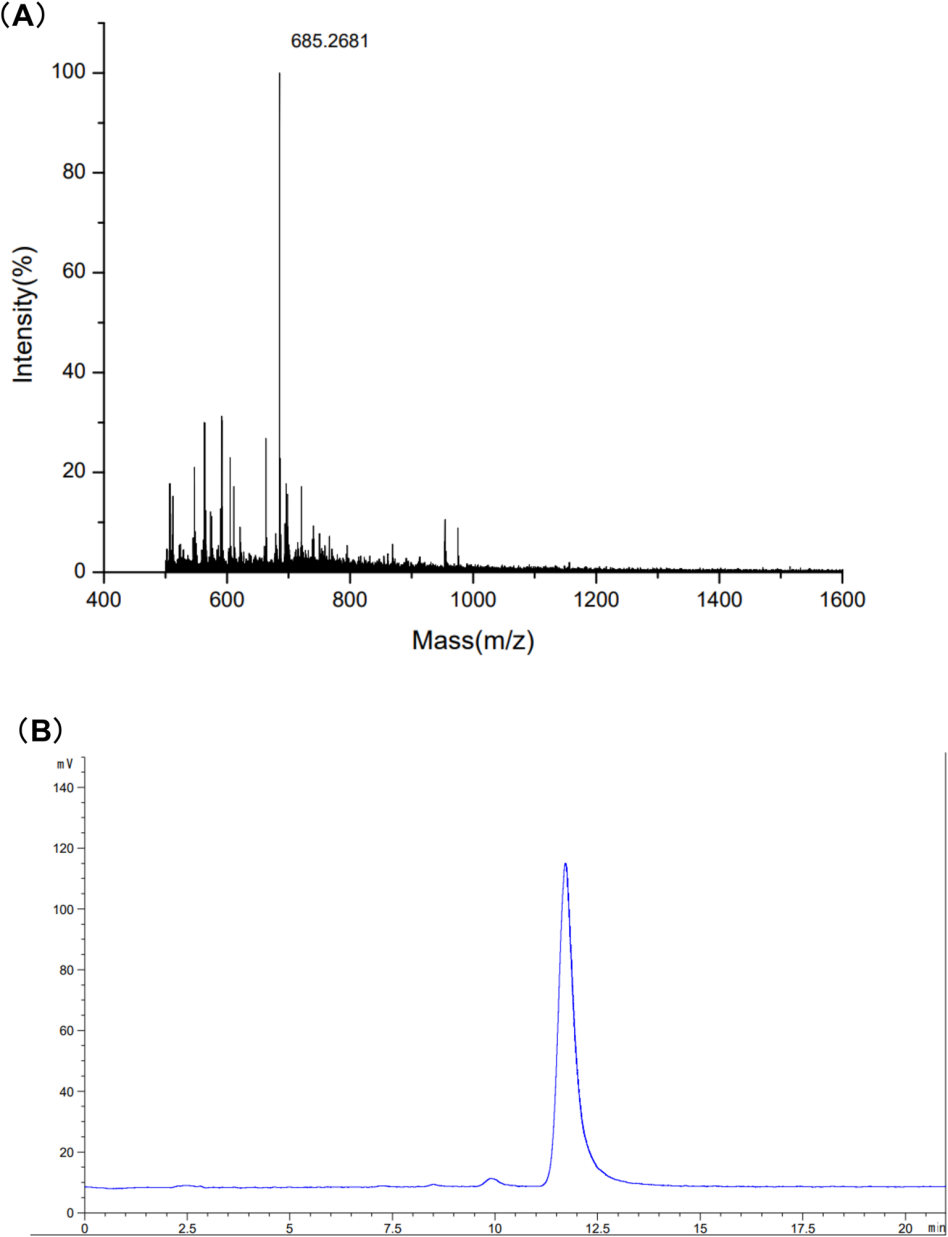
Structural characterization of the COS with a specific DP

The components of the singular-DP COS are analyzed and presented in Fig. 2. The MALDI-TOF-MS results showed that the COS mass-to-charge ratio (*m*/*z*) was 685.2681 mass units, which was consistent with its [M+Na+] ion-peak, displaying a DP of 4. The COS purity was identified via HPLC (Fig. 2), showing one peak and a retention time of 11.6 min. The findings revealed a high COS purity and degree of deacetylation.

### Establishment of the diabetes model

After 8 weeks of high-fat feeding, the mice were continuously injected with small doses of STZ, resulting in obesity, lower glucose tolerance, IR, and symptoms of polydipsia and polyphagia. At 14 days, the random blood glucose value of the mice exceeded 16.5 mol/L (Additional file 2: Figure S1), successfully establishing the diabetes model.

**Fig. 3:**
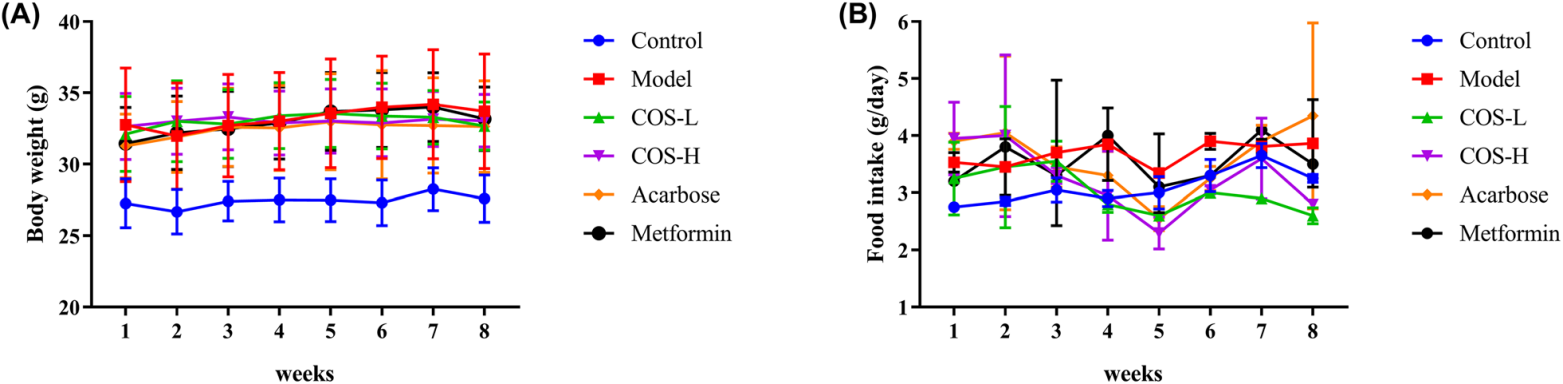
Changes in the **A** body weight and **B** food intake during the intervention process

The model group (*p* < 0.05) body weight value was considerably higher than the normal group, as shown in Fig. 3, while no substantial changes were apparent in the body weight and food intake levels of the intervention groups. Table 1 shows that the weight of the pancreas (*p* < 0.05) and muscles (*p* < 0.05) of the model group were significantly lower than the normal group, while the weight of the fat (*p* < 0.05) was higher. However, no pronounced differences were evident between the intervention and the model groups regarding the organ indexes.

**Table 1:** The effect of COS on the tissue weight of the mice

Index	Control	Model	COS-L	COS-H	Acarbose	Metformin
Pancreas (%BW)	0.59 ± 0.14^a^	0.48 ± 0.10^c^	0.52 ± 0.07^b^	0.63 ± 0.12^a^	0.56 ± 0.06^a^	0.50 ± 0.06^b^
Muscle (%BW)	4.72 ± 1.02^a^	3.88 ± 0.35^b^	3.90 ± 0.40^b^	3.84 ± 0.46^b^	3.94 ± 0.34^b^	3.64 ± 0.49^b^
Liver (%BW)	1.36 ± 0.23^a^	1.17 ± 0.21^c^	1.22 ± 0.28^b^	1.22 ± 0.21^b^	1.39 ± 0.14^b^	1.26 ± 0.09^b^
Epididymal fat (%BW)	1.17 ± 0.22^b^	3.30 ± 0.90^a^	2.72 ± 0.85^a^	3.54 ± 0.89^a^	2.33 ± 0.93^a^	3.74 ± 0.63^a^
Kidney (%BW)	0.65 ± 0.11^a^	0.51 ± 0.08^b^	0.53 ± 0.11^b^	0.51 ± 0.09 ^b^	0.46 ± 0.03^b^	0.38 ± 0.04^b^
Spleen (%BW)	0.28 ± 0.02^a^	0.27 ± 0.06^c^	0.29 ± 0.04b^c^	0.28 ± 0.05b^c^	0.29 ± 0.03^b^	0.25 ± 0.04^bc^
Heart (%BW)	1.11 ± 0.10^a^	1.14 ± 0.14^d^	1.20 ± 0.09^b^	1.17 ± 0.06^b^	1.25 ± 0.09^bc^	1.07 ± 0.07^cd^

Different letters in the same column represent significant differences between the treatments when *p* < 0.05

### COS improved the IR and increased the insulin sensitivity of the T2DM mice

**Fig. 4:**
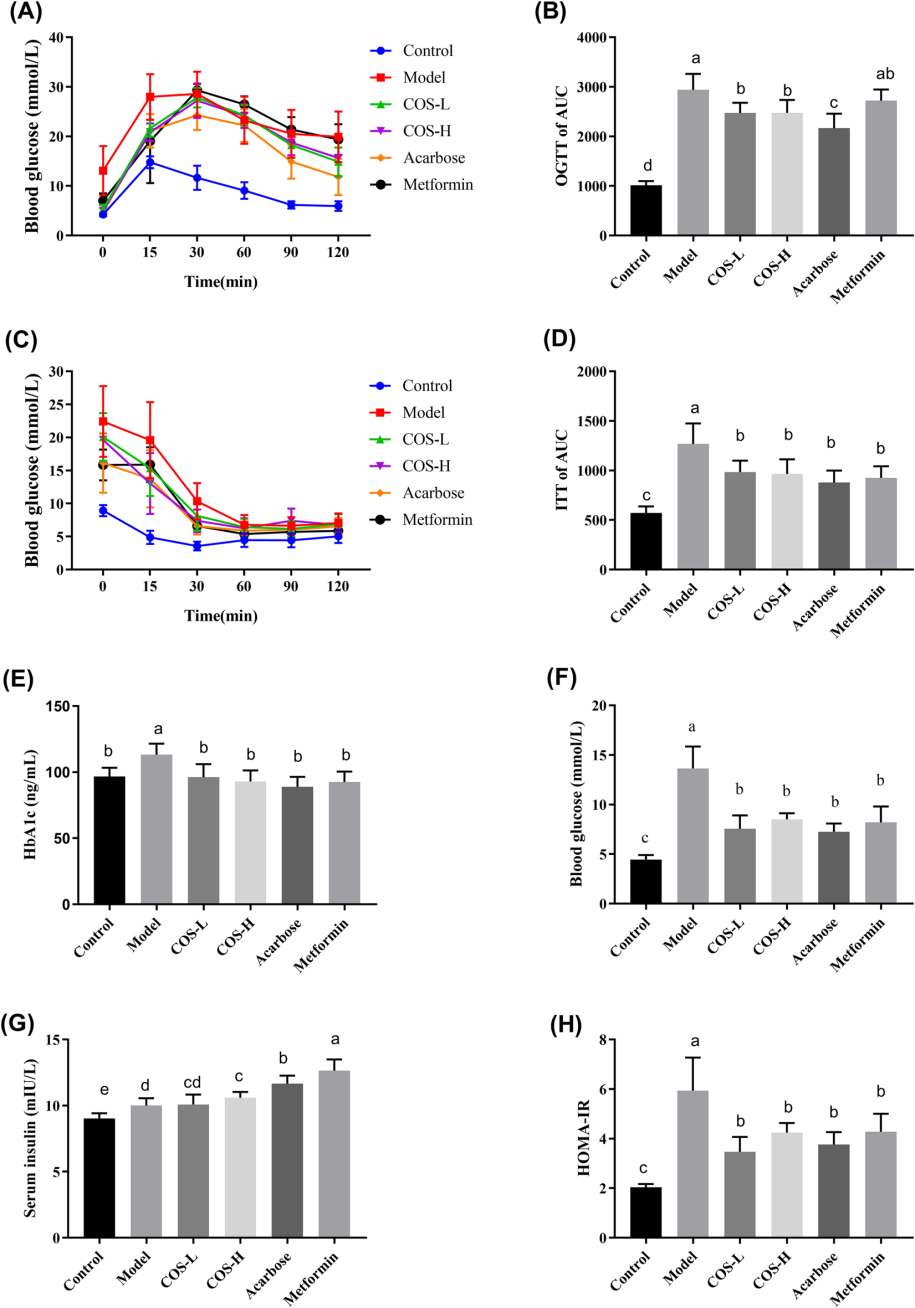
The impact of COS on the insulin and glucose tolerance of the mice. **A** OGTT, **B** glucose AUC, **C** ITT, **D** insulin AUC, **E** HbA1c levels, **F** blood glucose level, **G** levels of serum insulin, **H** HOMA-IR index

The OGTT and ITT are essential indicators when assessing glucose metabolism and monitoring glucose tolerance. The glucose AUC of the COS group was substantially lower (*p* < 0.05) than the diabetes model group, suggesting that COS enhanced the insulin sensitivity of T2DM mice (Fig. 4A, B). Furthermore, the blood glucose and AUC of the mice in the diabetes model group exceeded the values in the normal group (*p* < 0.05) after insulin injection (Fig. 4C, D). The blood glucose and insulin AUC of the mice treated with COS decreased faster (*p* < 0.05) than in the diabetes model group, suggesting that COS increased the utilization efficiency of insulin and improved insulin sensitivity.

Fasting glucose and insulin levels are vital indicators when measuring insulin sensitivity. Although the diabetic mice exhibited higher insulin levels (*p* < 0.05) (Fig. 4G), the fasting blood glucose levels still exceeded those in the normal group (*p* < 0.05), as shown in Fig. 4F. High insulin levels could not lower the glucose level in the blood, indicating a decrease in the insulin sensitivity of the diabetic mice. Although the COS group displayed a higher insulin level, the fasting blood glucose was substantially lower than the diabetic group (*p* < 0.05), showing that COS restored insulin sensitivity. Glucose and insulin concentration information were used to determine the HOMA-IR index. The results showed that the HOMA-IR level was substantially lower in the COS group (*p* < 0.05) than in the model group, as illustrated in Fig. 4H. Moreover, the serum glycosylated hemoglobin (HbA1c) level (Fig. 4E), which is used as a monitoring indicator for diabetic blood glucose control, returned to normal levels after COS intervention. These results indicated that COS regulated the fasting blood glucose levels, reduced IR, and increased insulin sensitivity.

### COS improved blood lipid levels and reduced liver lipid accumulation

**Fig. 5:**
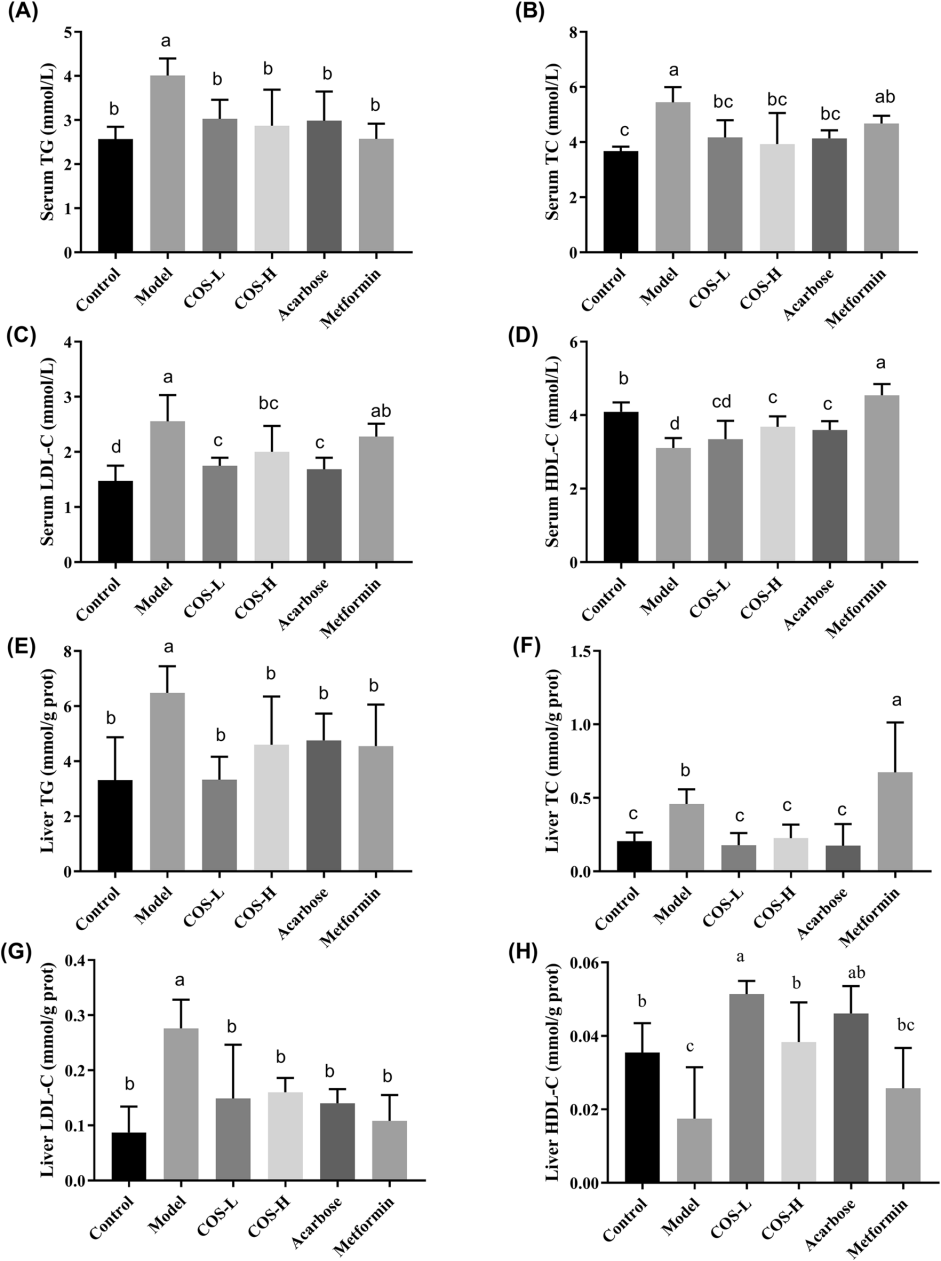
The effect of COS on the biochemical serum and liver indexes of the mice. **A** TG level in the serum. **B** TC level in the serum. **C** LDL-C level in the serum. **D** HDL-C level in the serum. **E** TG level in the liver. **F** TC level in the liver. **G** LDL-C level in the liver. **H** HDL-C level in the liver

The occurrence of diabetes is usually related to abnormal lipid metabolism. The LDL-C, TC, and TG levels in the serum and liver of the diabetes model group were markedly higher (*p* < 0.05, all) than in the normal group, while the HDL-C was substantially lower (Fig. 5). Compared with the model group, COS administration significantly modulated the blood and hepatic lipid profiles, indicating that COS improved the IR caused by lipid metabolism disorder.

### COS inhibited digestive enzyme activity and delayed carbohydrate absorption

**Fig. 6:**
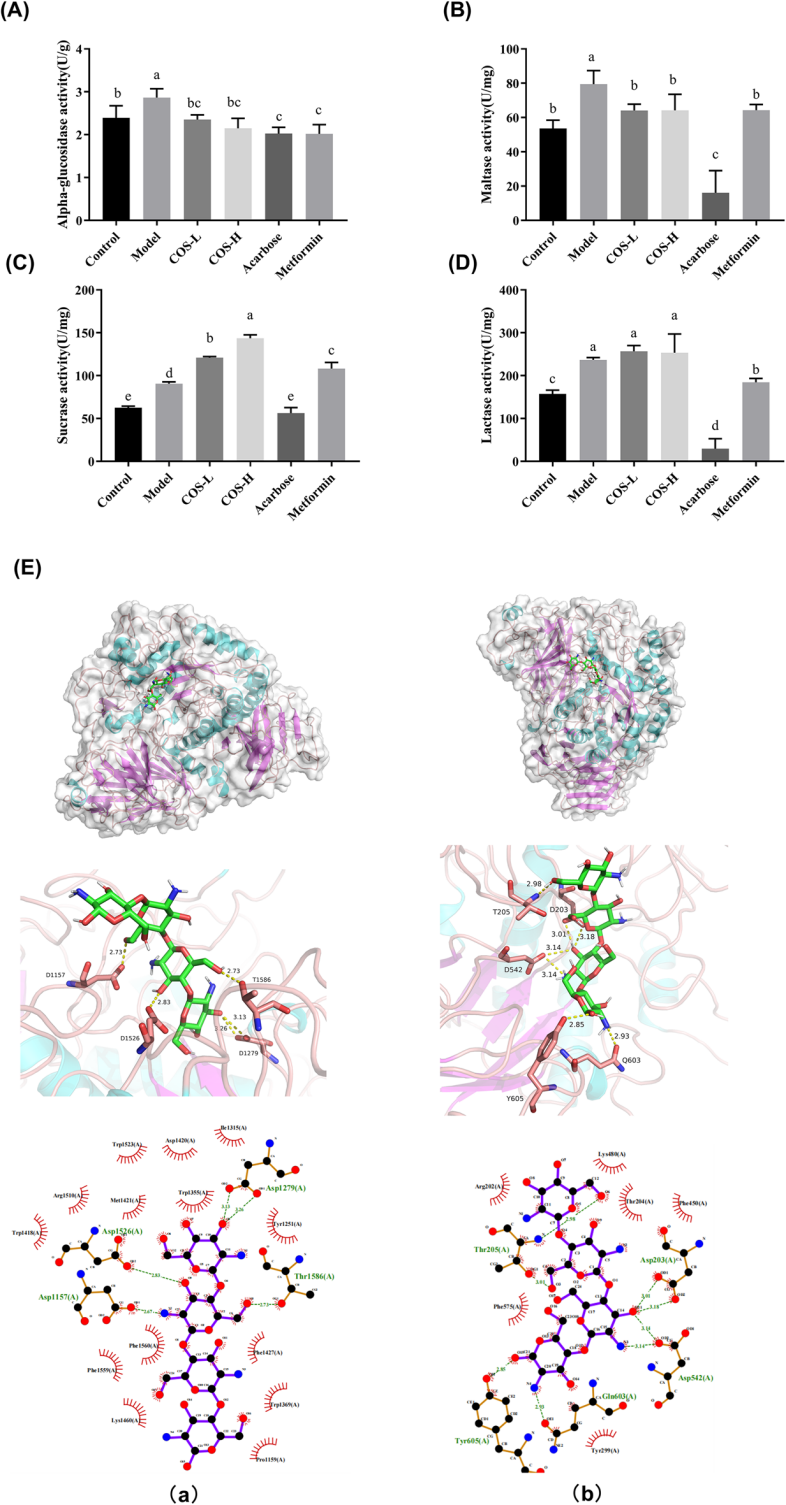
**A** The effect of COS on the α-glycosidase activity, **B** the maltase activity, **C** the sucrase activity, **D** the lactase activity, and **E** molecular docking. A schematic diagram of the optimal conformational interaction between maltose amylase C and COS (Ea). A schematic diagram of the optimal conformational interaction between maltose amylase N and COS (Eb)

The small intestine represents the primary glucose absorption site (Riesenfeld et al. 1980). Since inhibiting glycosidase activity is crucial for delaying carbohydrate absorption in the small intestine and reducing postprandial blood glucose (Naik et al. 2014), the impact of COS on glycosidase activity was investigated. The alpha-glucosidase, maltase, sucrase, and lactase enzyme activity was measured to ascertain the effect of COS on intestinal digestive enzymes. The results showed that the COS group restricted alpha-glucosidase and maltase activity while displaying the optimal inhibitory effect on maltase (Fig. 6A–D). The structure–function relationship between maltase and COS was explored to further elucidate the inhibitory impact of COS on glycosidase activity. The COS and maltase formed a hydrophobic connection, as shown in Fig. 6. The docking between COS and the C-terminal of the maltase revealed a hydrogen bond interaction (Fig. 6Ea) involving amino acids, Asp1526 with a bond length of 2.83 Å, Asp1279 with bond lengths of 3.13 Å and 3.26 Å, Asp1157 with a bond length of 2.67 Å, and Thr1586 with a bond length of 2.73 Å. During the hydrogen interaction with the N-terminal (Fig. 6Eb), the maltase Thr205, Asp203, Asp542, Tyr605, and Gln603 formed bond lengths of 2.98 Å and 3.01 Å, 3.01 Å and 3.18 Å, 3.14 Å, 2.85 Å, and 2.93 Å, respectively. Therefore, the results indicated that the hydrogen bond interaction prompted the maltase and COS to form a stable complex that occupied functional enzymatic sites and reduced glucose absorption.

### COS inhibited islet cell apoptosis and restored pancreatic islet functionality

**Fig. 7:**
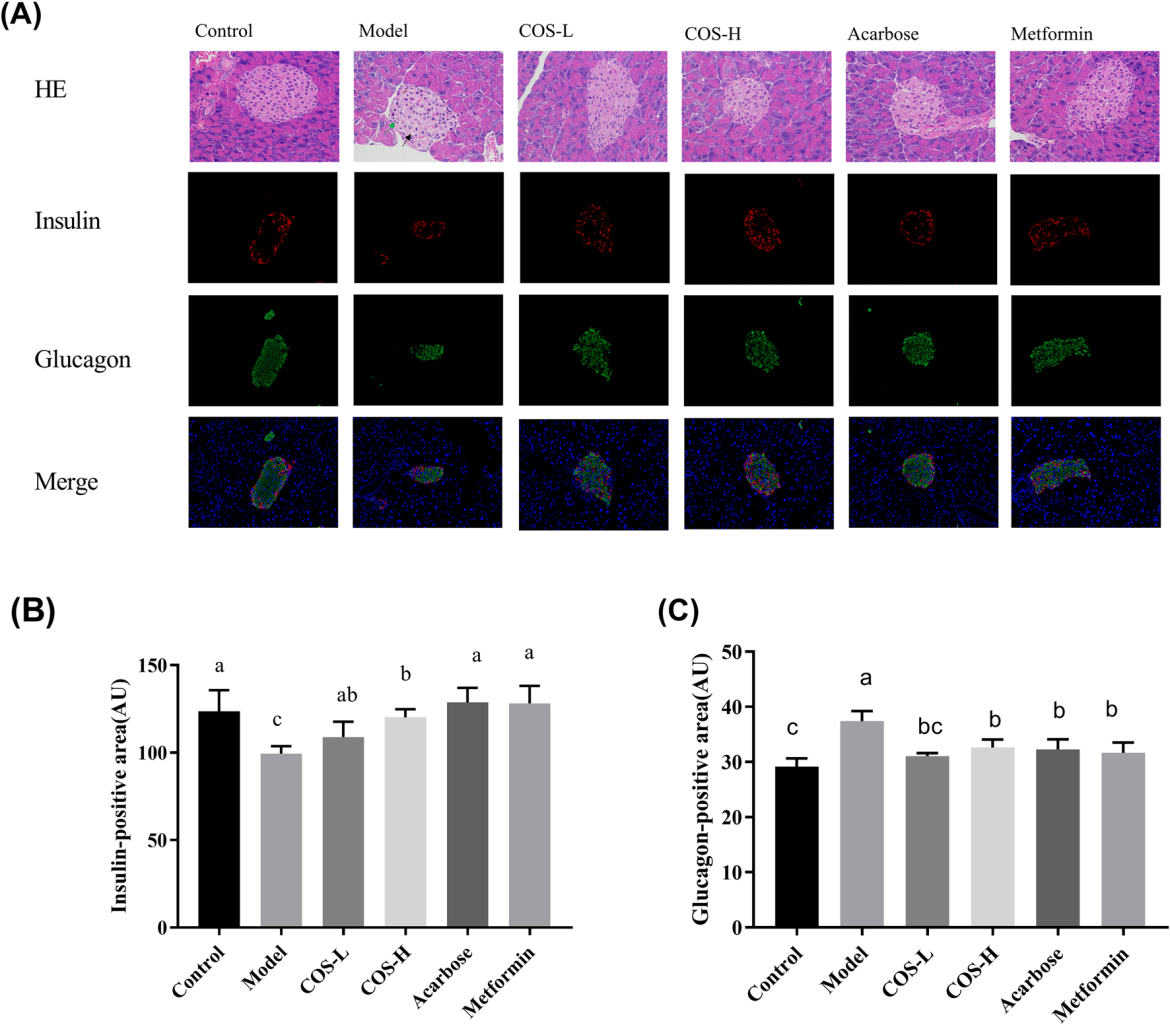
The **A** HE and immunofluorescence staining of the mouse pancreas and the fluorescence intensity of the **B** insulin and **C** glucagon in the pancreas of the mice

Since insufficient insulin secretion and islet cell apoptosis play a vital role in diabetic pathogenesis (Weir and Bonner-Weir 2004), the impact of COS on pancreatic functionality was explored. The HE staining results of the pancreas are shown in Fig. 7, indicating that the pancreatic islets in the model group were severely damaged, exhibiting atrophied pancreatic islet cells. Fewer islet cells were evident, and the cytoplasm was atrophic (black arrow). The islet numbers were restored, and the islet structure recovered after COS intervention. Islet damage can lead to islet cell dysfunction, affecting normal insulin secretion. Immunofluorescence staining of the insulin and glucagon showed that the insulin secretion was up-regulated, while glucagon secretion was down-regulated in the COS group. This suggests that COS promotes insulin secretion, maintains blood sugar balance, and improves pancreatic islet cell functionality, as shown in Fig. 7.

**Fig. 8:**
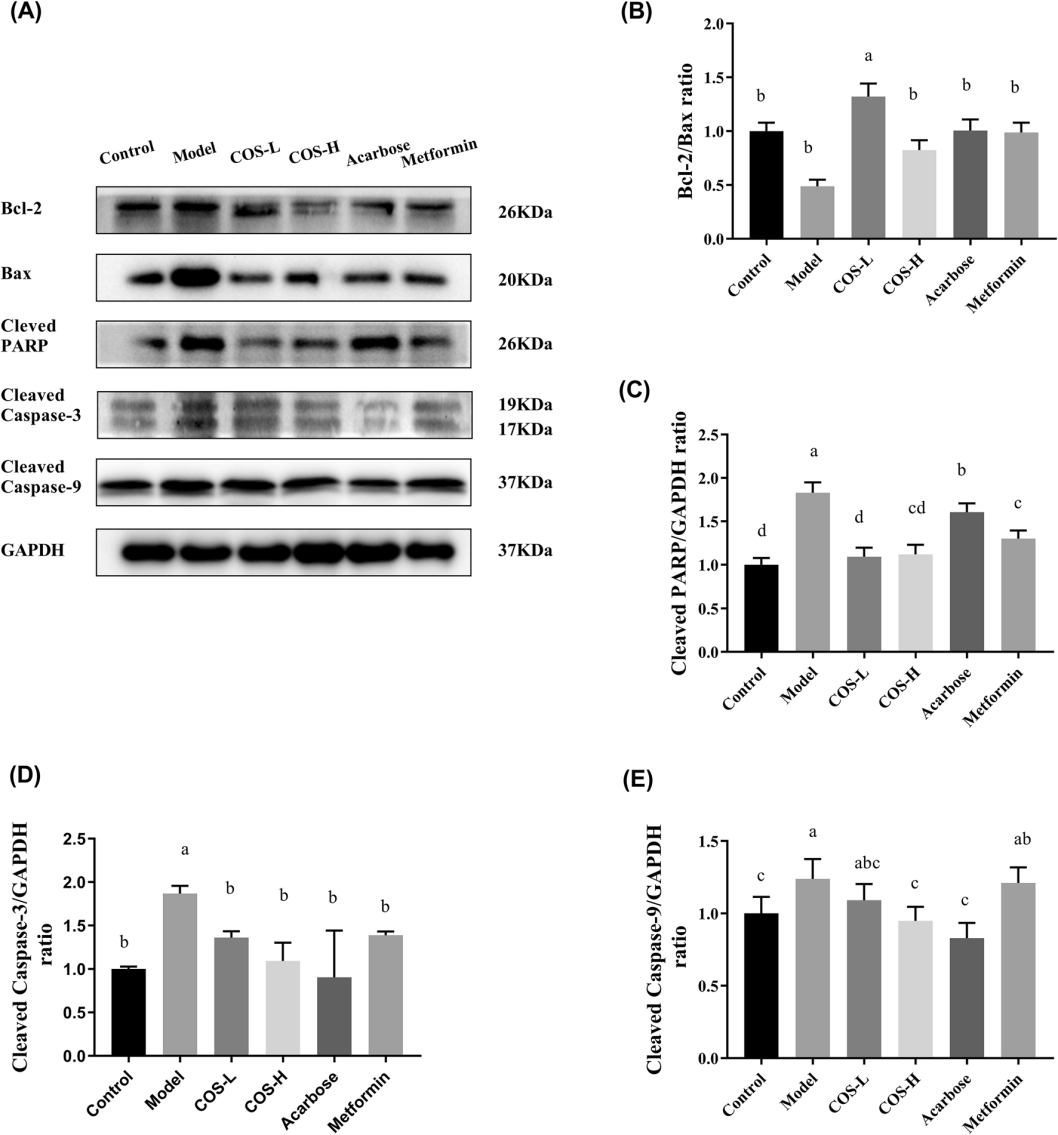
The apoptotic protein expression in the mouse pancreas. **A** Western blot images, **B** Bcl2/Bax, **C** cleaved PARP/GAPDH, **D** cleaved caspase-3/GAPDH, **E** cleaved caspase-9/GAPDH. Normalization versus GAPDH ensured equal protein loadings

The impact of COS on the apoptotic protein expression in the pancreas was examined to further investigate the underlying mechanisms. Moreover, the expression levels of the apoptotic protein of cleaved caspase-9, cleaved caspase-3, and cleaved PARP were significantly higher in the model group than in the control group, while the Bcl-2/Bax ratio displayed a substantial decrease. COS intervention reduced the cleaved PARP, cleaved caspase-9, and cleaved caspase-3 levels while markedly increasing the Bcl-2/Bax ratio (Fig. 8). These results demonstrated that COS repaired T2DM-induced islet cell injury and restored insulin secretion.

### COS sustained glucose homeostasis and regulated the metabolism of glucose

**Fig. 9:**
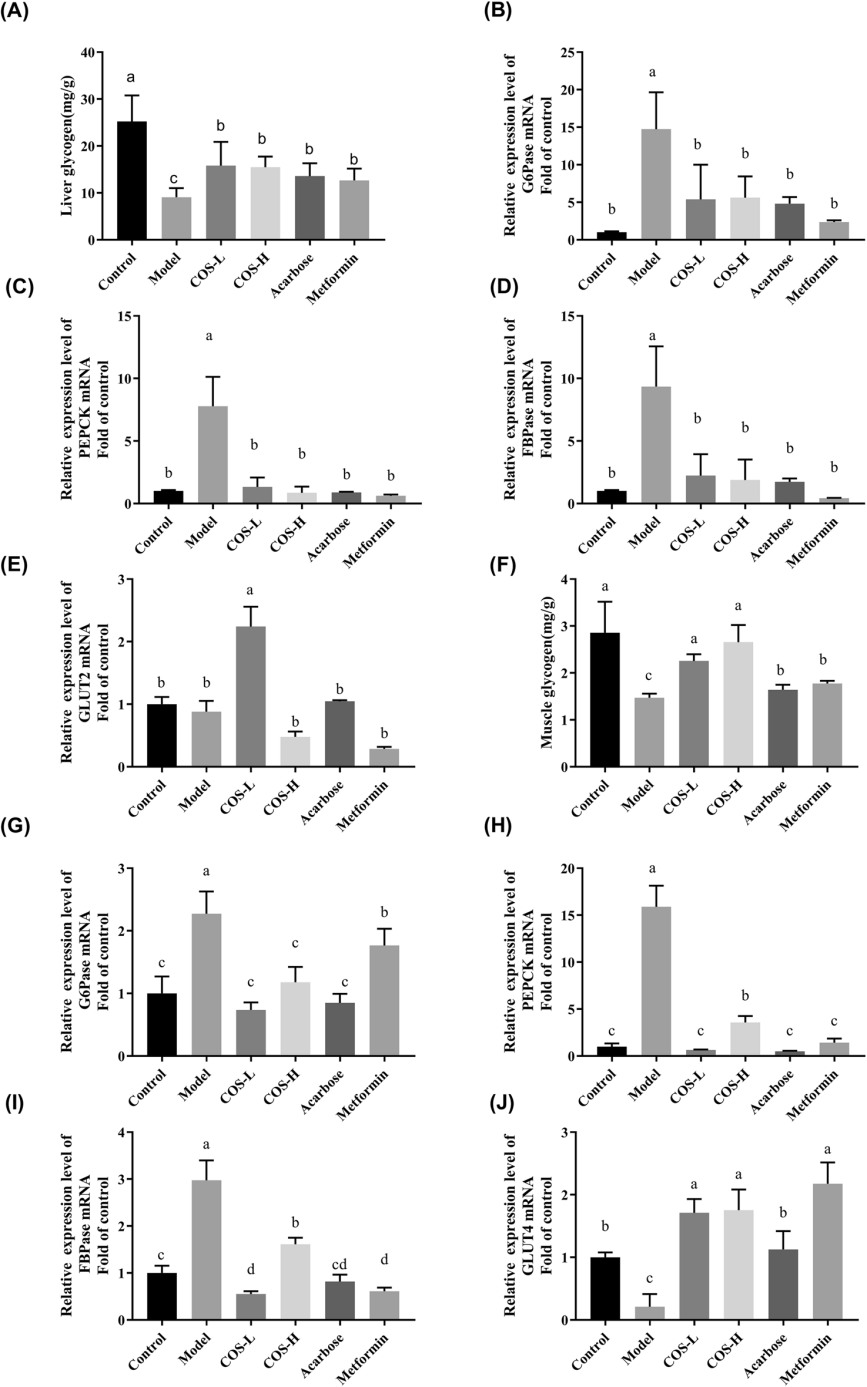
The effect of COS on the mRNA expression of the glucose metabolism indexes in the mice. **A** Glycogen content in the liver, **B** liver G6Pase, **C** liver PEPCK, **D** liver FBPase, **E** liver GLUT2, **F** glycogen content in the skeletal muscles, **G** muscle G6Pase, **H** muscle PEPCK, **I** muscle FBPase, **J** muscle GLUT4

**Fig. 10:**
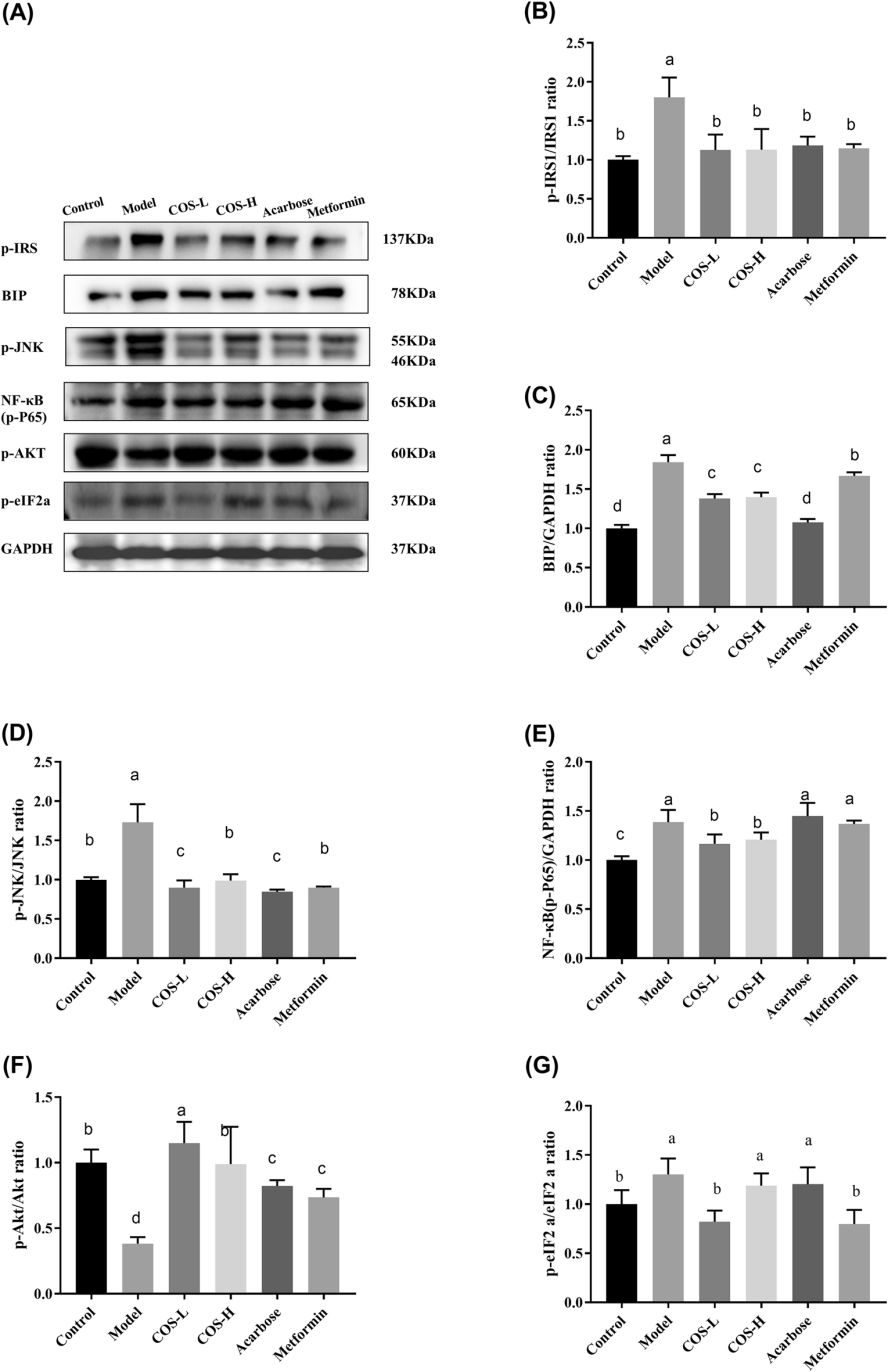
The expression of the proteins related to the insulin signaling pathway in the mouse livers. **A** Western blot images, **B** P-IRS-1 (Ser307)/GAPDH, **C** BIP/GAPDH, **D** p-JNK (Thr183 + Tyr185)/GAPDH, **E** NF-κB (P-p65) (Ser536)/GAPDH, **F** p-Akt (Ser473)/GAPDH, **G** p-eIF2A (Ser52)/GAPDH. Normalization versus GAPDH ensured equal protein loadings

IR in the skeletal muscles and liver is considered pathogenic of T2DM (Samuel and Shulman 2016). The sensitivity of the liver and muscles to insulin is reduced, causing glucose metabolism disorders in these areas and eventually hyperglycemia (Meynial-Denis et al. 2005). Therefore, to verify the effect of COS on glucose metabolism, the glycogen content in the liver and muscles, as well as the related gene expression levels during glucose metabolism signaling, were investigated. The muscle (Fig. 9F) and liver (Fig. 10A) glycogen content was considerably lower in the model group (*p* < 0.05) than in the control group, while the mRNA levels expressed by the main gluconeogenic enzymes, such as G6Pase, FBPase, and PEPCK were substantially up-regulated (*p* < 0.05) in the liver and skeletal muscles (Fig. 9). The mRNA level of GLUT2 in the liver displayed no significant changes (Fig. 9E), while the mRNA level of GLUT4 in the muscles showed a substantial decrease (*p* < 0.05) (Fig. 9J), suggesting abnormal glucose metabolism in the model group (Fig. 9). Although the liver and muscle glycogen content substantially exceeded (*p* < 0.05) that in the model group after COS intervention, the mRNA levels of G6Pase, FBPase, and PEPCK (*p* < 0.05) in the liver and skeletal muscles were lower (Fig. 9). In addition, compared with the model group (Fig. 9E), treatment with low-dose COS up-regulated (*p* < 0.05) the mRNA expressed by GLUT2 in the liver, while increasing GLUT4 mRNA expression in the skeletal muscles (*p* < 0.05) (Fig. 9J). These results indicated that COS enhanced glucose transshipment and usage by the liver and skeletal muscles, facilitating glycogen synthesis and reducing IR.

### COS decreased the response to ER stress and improved insulin signal transduction

**Fig. 11:**
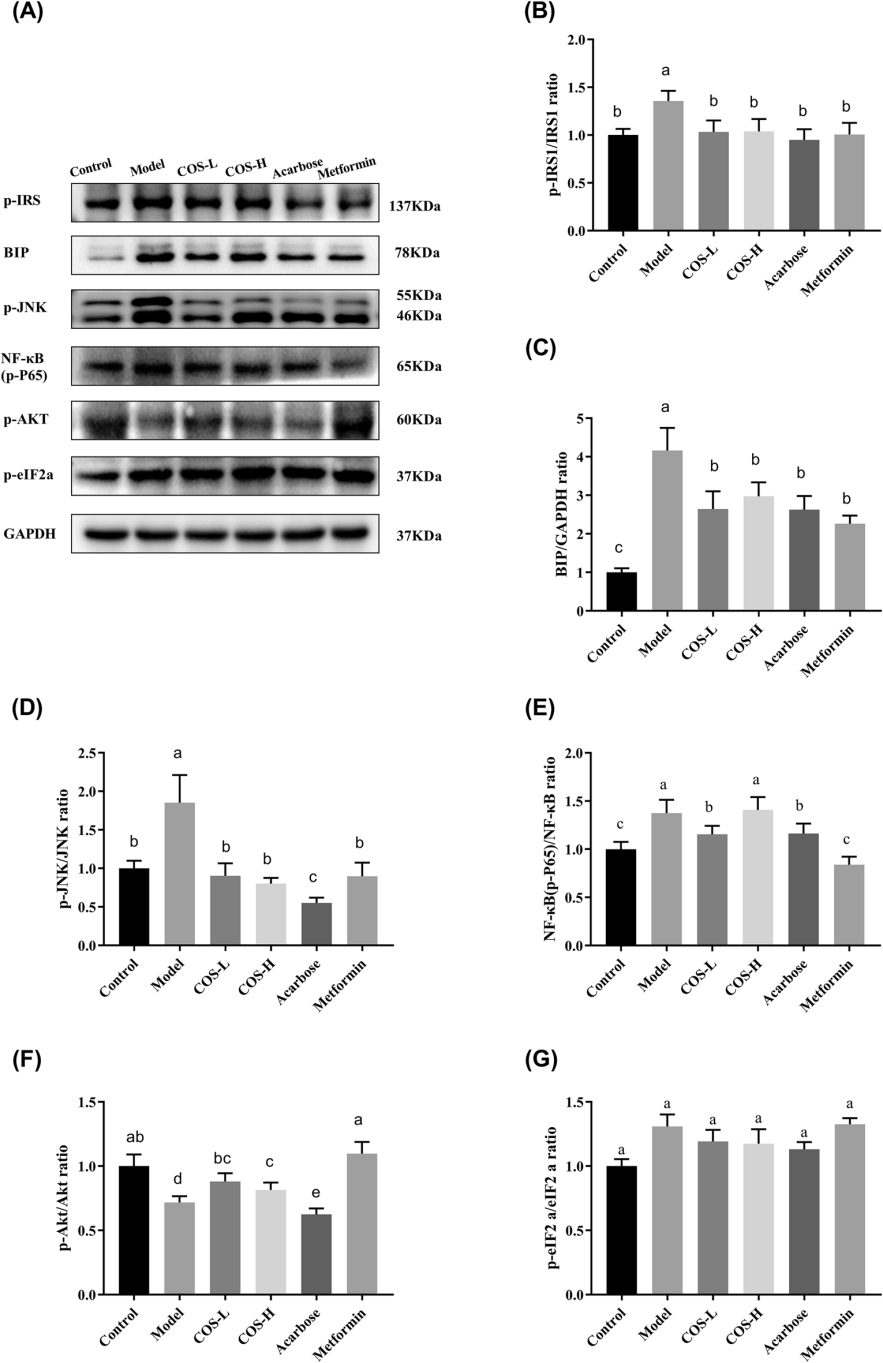
The expression of proteins related to the insulin signaling pathway in the mouse muscles. **A** Western blot images, **B** P-IRS-1 (Ser307)/GAPDH, **C** BIP/GAPDH, **D** p-JNK (Thr183 + Tyr185)/GAPDH, **E** NF-κB (P-p65) (Ser536)/GAPDH, **F** p-Akt (Ser473)/GAPDH, **G** p-eIF2A (Ser52)/GAPDH. Normalization versus GAPDH ensured equal protein loadings

**Fig. 12:**
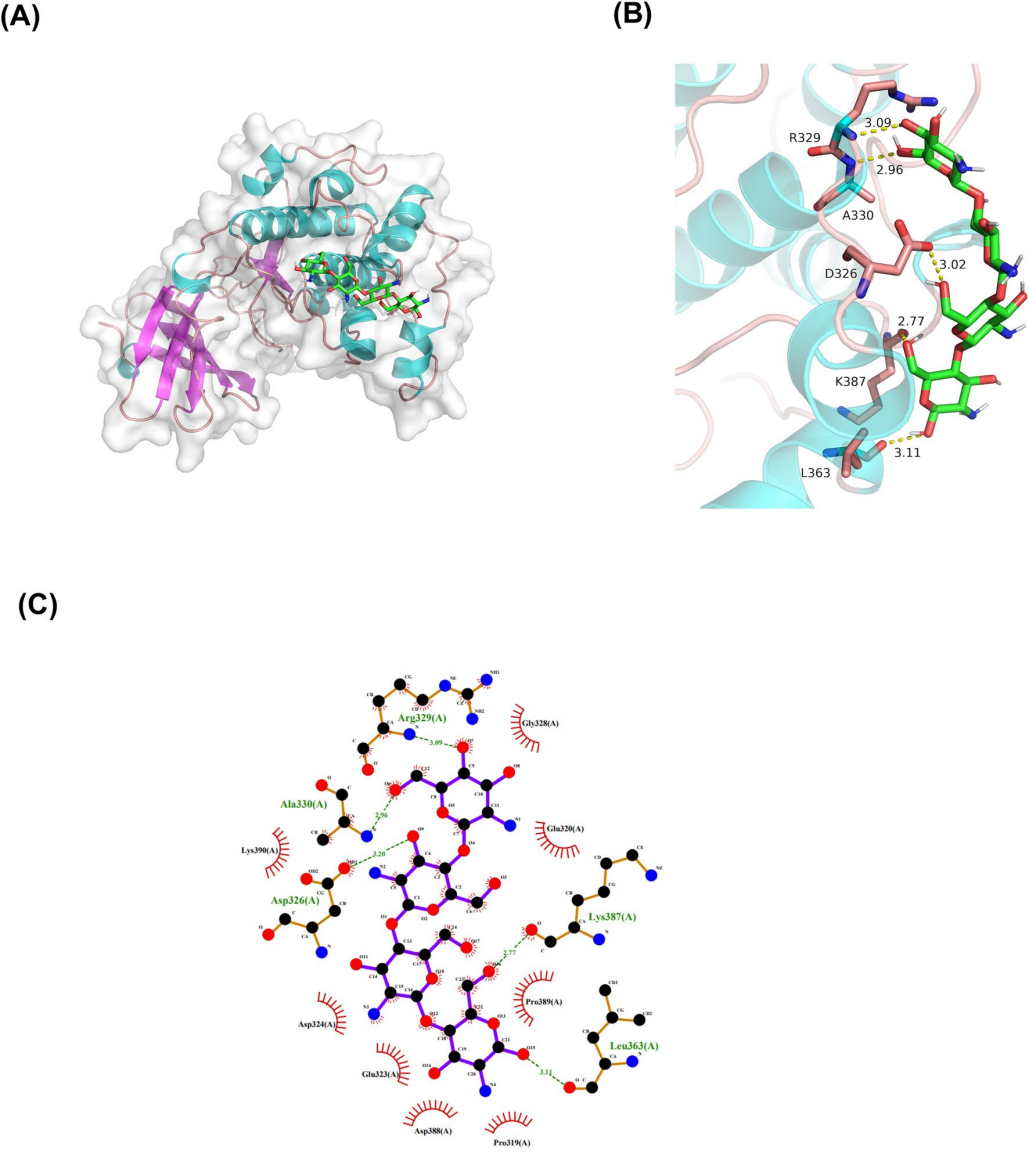
The predicted optimal binding mode of the COS docked with Akt/PKB. **A** Surface representation, **B** details of the interactions, **C** 2D interaction diagrams

Insulin signaling dysfunction affects normal insulin sensitivity, causing IR. This study evaluated the changes in the insulin signaling molecule-related genes and proteins in the mouse tissues to determine how COS improved insulin signal transduction (Figs. 10 and 11). The results showed that the p-IRS-1 (Ser307) was significantly lower in the normal group than in the model group, exhibiting a value of *p* < 0.05 (Figs. 10B and 11B), while the p-AKT phosphorylation level (Ser473) (Figs. 10F and 11F) was considerably lower than in the control group (*p* < 0.05), suggesting that the insulin signal transduction was impaired. The p-IRS-1 (Ser307) (Figs. 10B and 11B) expression was lower than in the model group, while p-AKT (Ser473) (Figs. 10F and 11F) expression increased after COS intervention. Moreover, the docking data suggested that the binding of COS to AKT involved six hydrogen bond formation processes (Arg329, Ala330, Asp326, Lys387, and Leu363) (Fig. 12). The free binding energy of COS and AKT was − 5.8 kcal/mol. This data suggests that COS may allosterically activate the kinase, which is conducive to AKT phosphorylation (Yang et al. 2002).

ER stress is vital in IR development, and its presence in peripheral tissues restricts insulin signaling via signaling cascade activation (Salvadó et al. 2015). The BIP expression and phosphorylation levels of p-JNK (Thrl83/Tyrl85), p-NF-KB p65 (Ser536), and p-eIF2a (Ser52) were substantially lower in the normal group than in the model group (Figs. 10 and 11), suggesting the activation of ER stress. COS intervention improved the phosphorylation level and BIP expression (*p* < 0.05) of p-IRS-1 (Ser307), p-JNK (Thrl83/Tyrl85), p-NF-KB p65 (Ser536), and p-eIF2a (Ser52) in the liver (Fig. 10). In muscle (Fig. 11), both high and low COS doses reduced the p-IRS-1 (Ser307) and p-JNK (Thrl83/Tyrl85) levels (*p* < 0.05), while only low COS doses reduced the p-NF-κB p65 (Ser536) (*p* < 0.05). Furthermore, neither high nor low COS doses could reduce the phosphorylation level of p-eIF2a (Ser52). Therefore, the reduction of ER stress and the attenuation of the insulin signaling pathway contributes to the hyperglycemic impact of COS.

## Discussion

T2DM is a multi-factor metabolic syndrome, mainly characterized by chronic hyperglycemia due to a reduced sensitivity to insulin and impaired insulin secretion, leading to multiple organ dysfunction (Chatterjee et al. 2017). Chitooligosaccharide is a food-derived natural product; its safety has been tested. The safety dose of COS reaches 400 mg/kg/day (He et al. 2020). Previous studies have shown that COS displays beneficial biological activity and hypoglycemic effects (Muanprasat and Chatsudthipong 2017; Naveed et al. 2019; Sutthasupha and Lungkaphin 2020). The hypoglycemic mechanism of COS mainly included the protection of pancreatic islet cells (Karadeniz et al. 2010) and improved intestinal absorption and transport (Yu et al. 2017). In addition, lipid metabolism (Bai et al. 2018; Li et al. 2018) improvement and intestinal microbiota (Wang et al. 2020) balance were also considered. However, since most of these studies used COS mixtures with different DPs, this study utilized a singular-DP COS to investigate the intervention mechanism while highlighting its anti-diabetic impact on multiple targets, such as the intestine, pancreas, liver, and skeletal muscles in vivo. This research hopes to develop a new anti-diabetic, biologically active substance.

In this study, the T2DM mice showed obvious symptoms of hyperglycemia, glucose intolerance, IR, and lipid metabolism disorders. COS intervention distinctly decreased the glycosylated hemoglobin and fasting glucose levels in the blood while improving insulin and glucose tolerance (Fig. 4). Additionally, COS improved insulin sensitivity depending on the HOMA-IR index (Fig. 4H). Furthermore, in terms of serum and liver lipid levels, COS also regulated lipid metabolites (TG, TC, LDL-C, and HDL-C) (Fig. 5), indicating its beneficial impact on diabetic hyperglycemia and hyperlipidemia.

The mechanism was explored further based on the phenotypes mentioned above. Due to the complexity of diabetic pathology, this study considered the intervention mechanism from aspects such as intestinal absorption, insulin secretion, and the target tissue utilization of glucose to clarify the COS mechanism and improve glucose homeostasis.

Carbohydrates represent the primary energy source for sustaining biological activities (Sajadimajd et al. 2019). They are decomposed into glucose by alpha-glucosidase in the intestine and absorbed by the blood for utilization by tissues and organs. Alpha-glucosidase denotes a family of enzymes (including maltase, sucrase, lactase, and amylase) directly involved in the glucose metabolism pathway (Ren et al. 2011). Inhibiting alpha-glucosidase activity in the intestine helps control blood glucose. In this study, the alpha-glucosidase activity (maltase, sucrase, and lactase) decreased after COS treatment, indicating that COS slowed down carbohydrate decomposition and reduced postprandial hyperglycemia (Fig. 6). Further molecular simulation showed that COS displayed hydrophobic interaction and hydrogen bonding with intestinal maltase, restricting effective carbohydrate and glycosidase consolidation and reducing glucose absorption.

The damage to islet beta cells and improper insulin secretion represent critical processes in diabetic pathogenesis. The T2DM mice exhibited destroyed pancreatic islet beta cells. The results of the HE staining, insulin immunofluorescence, and the expression analysis of apoptosis-related proteins indicated that COS had an excellent protective and recovery effect on pancreatic beta cells (Fig. 6).

The IR in the liver and skeletal muscles is primarily responsible for the development and occurrence of diabetes. Glucose absorption and storage via glycogen are balanced by the liver and skeletal muscles, restricting glucose release by preventing gluconeogenesis and glycogenolysis (Henriksen 2010; Achard and Laybutt 2012). The IR displayed by the T2DM mice prevented insulin signaling and the activation of downstream signaling molecules like IRS/AKT (Nandipati et al. 2017). Consequently, the liver and skeletal muscles failed to absorb and utilize the glucose, increasing the blood glucose levels. In this study, COS alleviated the T2DM mice glycogen reduction in the liver and skeletal muscles. Moreover, PEPCK, FBPase, and G6Pase represented the primary enzymes responsible for controlling glucose production (Lv et al. 2017; Wang and Dong 2019) in the IRS/AKT pathway to regulate the gluconeogenesis rate. COS down-regulated the PEPCK, FBPase, and G6Pase mRNA gene expression in the liver and skeletal muscles (Fig. 9), consequently promoting glycogen synthesis and inhibiting gluconeogenesis. Furthermore, the IRS-1 phosphorylation level decreased in the COS group while that of AKT increased, showing that COS improved insulin signal transduction (Figs. 10B and E and 11B and E). Additionally, the GLUT2 mRNA expression in the liver tissue was up-regulated by COS (Fig. 9E), while higher GLUT4 mRNA expression was evident in the skeletal muscles (Fig. 9J), illustrating that COS stimulated glucose transport and improved the glucose utilization rate. Therefore, COS can promote glucose uptake, reduce gluconeogenesis, and maintain glucostasis by activating the downstream molecules mediated by IRS1/AKT.

ER is essential in maintaining blood glucose homeostasis (Bánhegyi et al. 2007; Cnop et al. 2012; Flamment et al. 2012). Considering its role in the pathogenic status of T2DM, this study assessed the impact of COS on ER stress. The results revealed that COS markedly inhibited BIP and p-c-Jun expression (Figs. 10C, D and 11C, D) while restricting p-eIF2α and p-NF-κB expression (Figs. 10F, G and 11F, G) in T2DM mice. Therefore, COS protected diabetic mice from the damage associated with ER stress.

In conclusion, this study shows the multi-target modulation ability of COS, including inhibiting intestinal glucose absorption, accelerating glucose uptake and utilization in the liver and muscles, and improving pancreatic functionality, jointly regulating glucose metabolism in the body. Consequently, this study provides an extended scientific foundation for elucidating the anti-diabetic mechanism and distinct advantages of COS for developing new preventive or therapeutic medications for diabetes.

### Supplementary Information

**Additional file 1: Table S1.** Primer sequences used in qPCR.

**Additional file 2: Figure S1.** Establishment of the diabetes model. (A) Bodyweight. (B) Food intake. (C) OGTT. (D) AUC of the glucose. (E) Blood glucose.

## Data Availability

All data generated or analyzed during this study are included in this published article.

## Abbreviations

COS: Chitooligosaccharides
T2DM: Type 2 diabetes mellitus
IR: Insulin resistance
ER: Endoplasmic reticulum
elF2α: Eukaryotic translation initiation factor 2
JNK: C-Jun N-terminal kinase
IRS: Insulin receptor substrate
Akt: Protein kinase B
DP: Degree of polymerization
ITT: Insulin tolerance test
OGTT: Oral glucose tolerance test
TG: Total triglyceride
TC: Total cholesterol
LDL-C: Low-density lipoprotein
HDL-C: High-density lipoprotein
STZ: Streptozotocin
PEPCK: Phosphoenolpyruvate carboxykinase
FBPase: Fructose 1,6-bisphosphatase
G6Pase: Glucose-6-phosphatase
GLUT: Glucose transporter
BIP: Heavy-chain binding protein
