# Supplementary material for: Effect of chitooligosaccharides with a specific degree of polymerization on multiple targets in T2DM mice
Source: Bioresour Bioprocess. 2022 Sep 5;9(1):94. doi: 10.1186/s40643-022-00579-3 (PMC10992422; doi:10.1186/s40643-022-00579-3)
Supplement: Supplementary file 1 — Additional file 1: Table S1. Primer sequences used in qPCR. [file 40643_2022_579_MOESM1_ESM.docx]

Table S1 Primer sequences used in qPCR

| Gene | Forward (5’-3’) | Reverse (5’-3’) |
| --- | --- | --- |
| G6Pase | AGGAAGGATGGAGGAAGGAA | TGGAACCAGATGGGAAAGAG |
| PEPCK | GTTCCCAGGGTGCATGAAAG | AGGGCGAGTCTGTCAGTTCAA |
| FBPase | TGTGGGCTCCACCTGCCTGCACCTTTAGTC | TTTGATCGCGGTGCAGAGCGAATTCAGCAG |
| GLUT4 | ACCATAGGAGCTGGTGTGGTCAAT | GACCCATAGCATCCGCAACA |
| GLUT2 | GGGACTTGTGCTGCTGGATA | GAACACGTAAGGCCCAAGGA |
| GAPDH | CCTGAGCGCAAGTACTCTGTGT | GCTGATCCACATCTGCTGGAA |
