# Supplementary figures and images for: Effect of chitooligosaccharides with a specific degree of polymerization on multiple targets in T2DM mice
Source: Bioresour Bioprocess. 2022 Sep 5;9(1):94. doi: 10.1186/s40643-022-00579-3 (PMC10992422; doi:10.1186/s40643-022-00579-3)

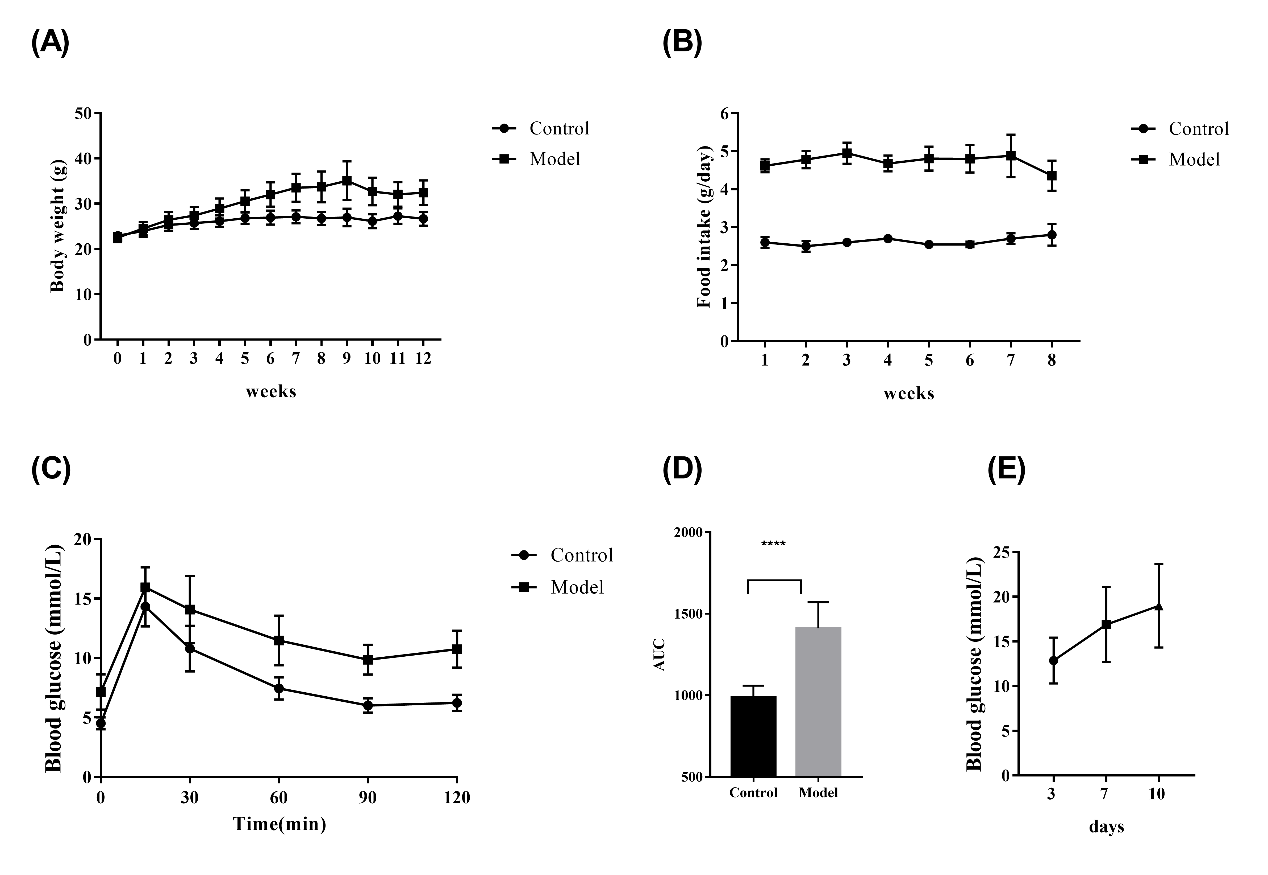

Supplement: Supplementary file 2 — Additional file 2: Figure S1. Establishment of the diabetes model. (A) Bodyweight. (B) Food intake. (C) OGTT. (D) AUC of the glucose. (E) Blood glucose. [file 40643_2022_579_MOESM2_ESM.tif]
